# Herpesvirus entry mediator on T cells as a protective factor for myasthenia gravis: A Mendelian randomization study

**DOI:** 10.3389/fimmu.2022.931821

**Published:** 2022-08-01

**Authors:** Huahua Zhong, Kexin Jiao, Xiao Huan, Rui Zhao, Manqiqige Su, Li-Ying Goh, Xueying Zheng, Zhirui Zhou, Sushan Luo, Chongbo Zhao

**Affiliations:** ^1^ Huashan Rare Disease Center and Department of Neurology, Huashan Hospital, Shanghai Medical College, Fudan University, National Center for Neurological Disorders, Shanghai, China; ^2^ Shanghai Medical College, Fudan University, Shanghai, China; ^3^ Department of Biostatistics, School of Public Health and Key Laboratory of Public Health Safety, Fudan University, Shanghai, China; ^4^ Radiation Oncology Center, Huashan Hospital, Fudan University, Shanghai, China

**Keywords:** Mendelian randomization, myasthenia gravis, GWAS, HVEM, T cell

## Abstract

**Background and objectives:**

Myasthenia gravis (MG) is a T cell-driven, autoantibody-mediated disorder affecting transmission in neuromuscular junctions. The associations between the peripheral T cells and MG have been extensively studied. However, they are mainly of observational nature, thus limiting our understanding of the effect of inflammatory biomarkers on MG risk. With large data sets now available, we used Mendelian randomization (MR) analysis to investigate whether the biomarkers on T cells are causally associated with MG and further validate the relationships.

**Methods:**

We performed a two-sample MR analysis using genetic data from one genome-wide association study (GWAS) for 210 extensive T-cell traits in 3,757 general population individuals and the largest GWAS for MG currently available (1,873 patients versus 36,370 age/gender-matched controls) from US and Italy. Then the biomarkers of interest were validated separately in two GWASs for MG in FIN biobank (232 patients versus 217,056 controls) and UK biobank (152 patients versus 386,631 controls).

**Results:**

In the first analysis, three T-cell traits were identified to be causally protective for MG risk: 1) CD8 on terminally differentiated CD8^+^ T cells (OR [95% CI] = 0.71 [0.59, 0.86], P = 5.62e-04, adjusted P =2.81e-02); 2) CD4^+^ regulatory T proportion in T cells (OR [95% CI] = 0.44 [0.26, 0.72], P = 1.30e-03, adjusted P =2.81e-02); 3) HVEM expression on total T cells (OR [95% CI] = 0.67 [0.52, 0.86], P = 1.61e-03, adjusted P =2.81e-02) and other eight T-cell subtypes (e.g., naïve CD4+ T cells). In particular, HVEM is a novel immune checkpoint on T cells that has never been linked to MG before. The SNPs on the TNFRSF14 *per se* further support a more direct link between the HVEM and MG. The validation analysis replicated these results in both FIN and UK biobanks. Both datasets showed a concordant protective trend supporting the findings, albeit not significant.

**Conclusion:**

This study highlighted the role of HVEM on T cells as a novel molecular-modified factor for MG risk and validated the causality between T cells and MG. These findings may advance our understanding of MG’s immunopathology and facilitate the future development of predictive disease-relevant biomarkers.

## Introduction

Myasthenia gravis (MG) is an autoimmune disease that mainly affects the postsynaptic membrane at the neuromuscular junction. Fatigability and weakness in skeletal muscles are the representing clinical features. Immune dysregulation in MG mainly involves malfunctioned T cells, autoreactive B cells, and autoantibody production (1). Autoantibodies that were against postsynaptic membrane components mainly consist of the anti-acetylcholine receptor (AChR), anti-muscle specific kinase (MuSK), and lipoprotein-related protein 4 (LRP4) antibodies (2).

The thymus is a gland where T cells differentiate and mature. The removal of thymus (thymectomy) brought long-term benefits by improving the clinical outcome in thymomatous and non-thymomatous MG patients (3, 4). In the immunological pathogenesis of AChR-associated MG, the thymus releases AChR autoreactive T cells to activate peripheral AChR-directed B cells (5). Besides, chronic inflammation maintained by circulating T helper 17 (Th17) cells, autoantibody production promoted by follicular T (Tfh) cells, and impaired rebalancing function of regulatory T (Treg) cells contribute to the MG exacerbation (6). In contrast, CD8^+^ T cells were involved in MG pathogenesis, and there are relatively very few studies investigating the exact correlations (7). Current studies on T cells and MG were mainly conventional and observational.

Mendelian randomization (MR) uses genetic variants as the exposure proxy of the exposure to examine the causal effect of that exposure on the outcome (8). The correlations between genetic variants and MG have been explored in several genome-wide association studies (GWASs) and human leukocyte antigen (HLA) haplotype analysis, by which T-cell relevant genes, including CTLA4, TNFRSF11A, PTPN22, and the HLA haplotypes, have been implicated in the pathogenesis of MG (9–12). With now available large data sets, MR analysis may be an elegant tool to explore the novel biomarkers from T cells with causal impacts on MG risk, which has rarely been performed in this field.

We hypothesized that molecules in peripheral T cell traits have direct causal effects on MG risk. A two-sample MR study was performed to determine this causal relationship by leveraging extensive T-cell traits from 3,757 general population-derived individuals and the largest GWAS on MG with 1,873 patients and 36,370 age- and gender-matched healthy controls. The results were further replicated in both FIN biobank with 217,288 individuals and UK biobank with 386,783 individuals. This study may establish causal links between the T-cell relevant molecules and MG development.

## Materials and methods

### Data sources

The current study applied a two-sample MR method to analyze causal relationships between 210 T-cell traits and MG. The data sources were chosen from studies with publicly available summary GWAS data, and detailed information about different GWAS datasets is displayed in **Table 1**. The extensive T-cell traits (listed in **Supplementary File 1**) were derived from the SardiNIA project composed of GWAS data from 3,757 general population individuals who are native to the central east coast of Sardinia, Italy (13). These T-cell traits included subtypes in the T-cell panel (double negative, double positive, CD4^+^, CD8^+^), regulatory T (Treg) panel, maturation stages (central memory/effector memory/terminally differentiated), and cell marker expression levels on different T cells. As a primary analysis, the MG data were sourced from the currently largest meta-GWAS conducted in the US and Italy (1,873 patients versus 36,370 age/gender-matched controls) (11). Only anti-acetylcholine receptor antibody-positive (AChR+) MG patients were enrolled in this study, and patients with positive test results for antibodies to muscle-specific kinase (MuSK+) were excluded from the enrollment. In the secondary analysis, the validation datasets include FIN Biobank (https://gwas.mrcieu.ac.uk/datasets/finn-b-G6_MYASTHENIA/) (232 patients versus 217,056 controls) and UK Biobank (http://www.nealelab.is/uk-biobank) (152 patients versus 386,631 controls). The MG phenotype was ascertained from participants’ self-reported questionnaires; information of MG subtypes is not applicable. All original studies obtained ethical approval and informed consent from the participants.

**Table 1:** GWAS datasets used in this Mendelian randomization (MR) study.

Dataset	Phenotype/variable	First author (year)	Sample size (cases/controls)	Population	Sex	Phenotype ascertainment
Exposure 1	210 kinds of T-cell traits and markers	Orrù (2020)	3,757	Sardinian (Italy)	57.0% female	Normal individuals’ peripheral blood was antibody-stained and processed for flow cytometry
Outcome 1	Myasthenia gravis	Chia (2022)	38,243 (1,873/36,370)	US and Italian	47.2% female	Patients diagnosed in myasthenia gravis clinics: characteristic fatigable weakness and electrophysiological and/or pharmacological abnormalities and confirmed by the presence of anti-acetylcholine receptor antibodies
Outcome 2		Fin biobank	217,288 (232/217,056)	Finnish	Mixed	Self-reported phenotype (myasthenia gravis subtype information are not applicable)
Outcome 3		UK biobank	386,783 (152/386,631)	UK	Mixed	Self-reported phenotype (myasthenia gravis subtype information are not applicable)

### Instrument selection

**Figure 1:**
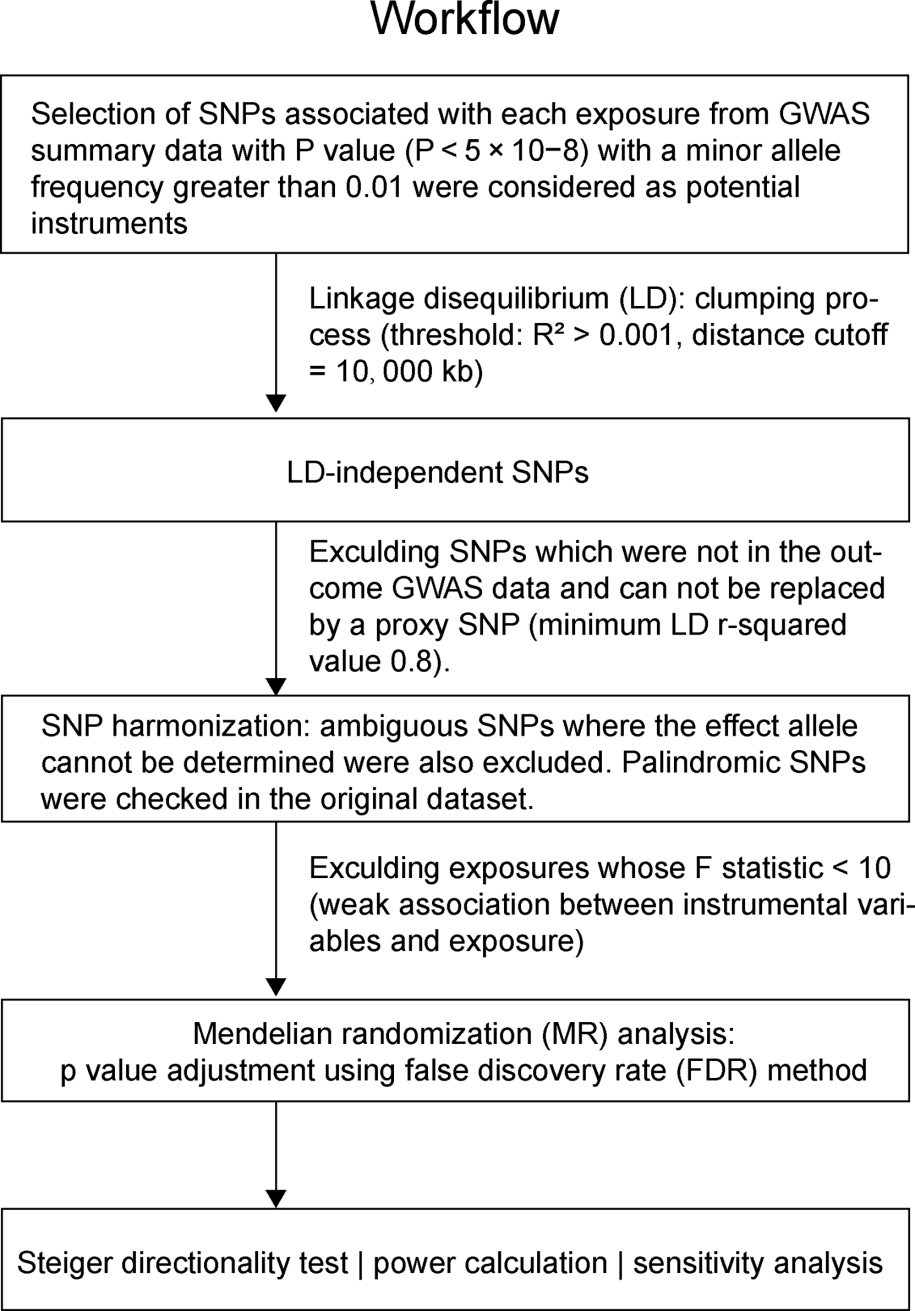
The workflow of instrumental SNP selection and Mendelian randomization (MR) analysis.

For selecting the most unbiased and representing instrumental genetic variables, a series of quality control steps were conducted to determine eligible instrumental SNPs (**Figure 1**). First, significant SNPs associated with exposures with genome-wide significance (P < 5 × 10^−8^) and minor allele frequency (MAF) > 0.01 were selected. Second, given that many SNPs may locate adjacently in linkage disequilibrium status in a GWAS, we performed a clumping process (R^2^ < 0.001, window size = 10,000 kb) using European reference samples from the 1000 genomes project and retained only the SNP with the lowest P-value. Third, exposure SNPs were extracted in the outcome GWAS summary data. If a particular exposure SNP was not present in the outcome GWAS, then a proxy SNP in linkage disequilibrium with the exposure SNP (minimum LD r-squared value 0.8) was used. Fourth, the exposure and outcome SNPs were harmonized, by which ambiguous SNPs in which the effect allele cannot be determined were removed. Palindromic SNPs were specifically checked in original datasets to avoid unwanted reverse effects. The strength of the genetic instrument was evaluated by F-statistics, and a weak instrument with F-statistic < 10 was removed. The calculation of the F statistic is F = R^2^(n-k-1)/k(1-R^2^), where R^2^ represents the exposure variance explained by the instrumental SNPs, n is the sample size, and k represents the number of instrumental variables (14). These stringently selected SNPs were used as the instrumental variables for the subsequent two-sample MR analysis.

### Two-sample MR analysis

Different MR methods were used to estimate the causative effect of exposure variables on the outcome accordingly. The Wald ratio method was used when only one instrumental SNP was available, and the inverse variance weighting (IVW) method was used when more than one SNP was presented. All causal estimates were converted to odds ratios (ORs) for the outcome which was a dichotomous phenotype. For exposure with more than three SNPs available, sensitivity analyses were performed using different MR methods which hold different assumptions at the cost of reduced statistical power, including weighted median (15), weighted mode (16), simple mode, MR Egger regression (17), and MR-PRESSO (18). The Steiger directionality test was performed in those significant results to validate whether the assumption that exposure causes outcome is valid (19). For exposures with less than three instrumental SNPs, pleiotropy analysis was performed using the PhenoScanner database to query additional associated traits found in previously published GWASs (20). Finally, statistical power for each exposure was calculated with a two-sided type-I error rate α = 0.05 (21).

### MR assumptions

Three core instrumental variable assumptions for this study were specifically considered: 1) Relevance: instrumental SNPs are associated with the exposure of T-cell signatures. The genetic bases for T-cell functions and subtypes have been fully investigated, and genetically engineered T-cell immunotherapies have provided remarkable clinical success (22). We also calculated the F-statistic for each T-cell signature, and only those instrumental SNPs with F-statistic > 10 were considered qualified. 2) Independence: there is no confounder between the instrumental SNPs and the outcome. Only genetic data sourced from European ancestry and both-sex populations were used in this study to avoid common confounders due to demographic variety. 3) Exclusion restriction: instrumental SNPs affect the outcome exclusively through their potential effects on the exposure T-cell signatures. The pathological mechanisms of how irregulated T cells cause MG have been explained in the introduction. To identify potential horizontal pleiotropy, we also searched the PhenoScanner database to find other impacts that might be caused by those instrumental SNPs.

### Statistical analysis

We performed the MR analyses in the R, version 4.1.2 (R Foundation for Statistical Computing, Vienna, Austria), with the TwoSampleMR package (23). Other packages used for processing data and generating figures include Tidyverse, Rsnps, and Forestplot. Since exposures (T-cell traits) were repeatedly compared with each outcome (MG), the P-values were adjusted by the false discovery rate (FDR) method.

## Results

The detailed characteristics of the instrumental SNPs associated with 210 T-cell traits (SNP n = 630) used in this study are displayed in **Supplementary File 1**. The MR findings between them and the outcome in each dataset are displayed in **Supplementary File 2**. The pleiotropy analysis results for those significant results are displayed in **Supplementary File 3**.

### Primary analysis: The US and Italian cohorts

**Figure 2:**
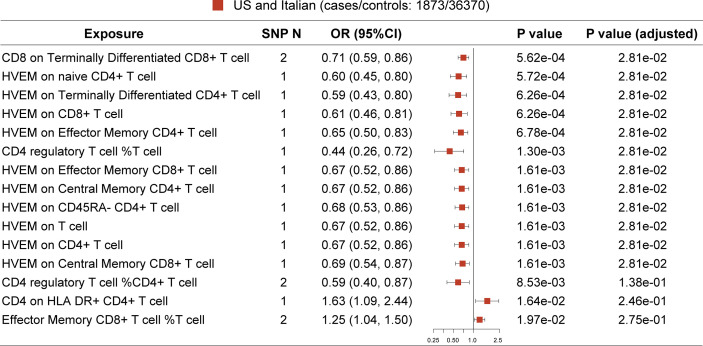
MR result in primary analysis (US and Italian patients). SNP N, number of SNP. The top 12 ranked T-cell traits by P value show protective effect on MG risk after false discovery rate (FDR) adjustment.

In the primary analysis, after FDR adjustment, the top 15 significant variables are as specifically displayed in **Figure 2**. All selected instrumental variants showed strong F statistics (median 223.24, IQR 1167.90) with the exposure, and the powers of all MR analyses were relatively large (median 1.00, IQR 0.03), as shown in **Table 2**. We identified three T-cell traits of interest which had protective effects on the risk of MG: 1) CD8 on terminally differentiated CD8^+^ T cells (OR [95% CI] = 0.71 [0.59, 0.86], P = 5.62e-04, adjusted P =2.81e-02); 2) CD4^+^ Tregs proportion in T cells (OR [95% CI] = 0.44 [0.26, 0.72], P = 1.30e-03, adjusted P =2.81e-02); 3) HVEM on total T cells (OR [95% CI] = 0.67 [0.52, 0.86], P = 1.61e-03, adjusted P =2.81e-02) and other eight T-cell subtypes (naive CD4^+^ T cells, terminally differentiated CD4^+^ T cells, CD8^+^ T cells, effector memory CD4^+^ T cells, CD4 regulatory T cells, effector memory CD8^+^ T cells, central memory CD4^+^ T cells, CD45RA^-^ CD4^+^ T cells). The Steiger directionality test showed that all results conformed to the right exposure to outcome direction.

**Table 2:** Detailed MR result in the primary analysis (the US and Italian cohorts).

Exposure	Method	SNP N	OR	r2.exposure	r2.outcome	P value (adjusted)	Power	F statistic	Correct causal direction	Steiger pval
CD8 on terminally differentiated CD8+ T cell	Inverse variance weighted	2	0.71	4.21E-02	3.11E-04	2.81E-02	0.83	82.46	TRUE	4.12E-23
HVEM on naive CD4+ T cell	Wald ratio	1	0.60	3.30E-02	3.11E-04	2.81E-02	0.98	128.21	TRUE	8.12E-09
HVEM on terminally differentiated CD4+ T cell	Wald ratio	1	0.59	5.61E-02	6.11E-04	2.81E-02	1.00	223.24	TRUE	5.59E-14
HVEM on CD8+ T cell	Wald ratio	1	0.61	6.83E-02	6.11E-04	2.81E-02	1.00	275.09	TRUE	3.59E-17
HVEM on effector memory CD4+ T cell	Wald ratio	1	0.65	4.15E-02	3.01E-04	2.81E-02	0.96	162.47	TRUE	5.15E-11
CD4 regulatory T cell %T cell	Wald ratio	1	0.44	2.00E-02	5.41E-04	2.81E-02	1.00	76.69	TRUE	2.25E-11
HVEM on effector memory CD8+ T cell	Wald ratio	1	0.67	2.42E-01	1.56E-03	2.81E-02	1.00	1196.25	TRUE	4.36E-67
HVEM on central memory CD4+ T cell	Wald ratio	1	0.67	2.55E-01	1.56E-03	2.81E-02	1.00	1286.80	TRUE	6.24E-72
HVEM on CD45RA- CD4+ T cell	Wald ratio	1	0.68	2.75E-01	1.56E-03	2.81E-02	1.00	1423.66	TRUE	3.66E-79
HVEM on T cell	Wald ratio	1	0.67	2.43E-01	1.56E-03	2.81E-02	1.00	1208.15	TRUE	1.00E-67
HVEM on CD4+ T cell	Wald ratio	1	0.67	2.66E-01	1.56E-03	2.81E-02	1.00	1362.65	TRUE	5.93E-76
HVEM on central memory CD8+ T cell	Wald ratio	1	0.69	2.96E-01	1.56E-03	2.81E-02	1.00	1578.89	TRUE	3.11E-87
CD4 regulatory T cell %CD4+ T cell	Inverse variance weighted	2	0.59	3.81E-02	5.76E-04	1.38E-01	0.99	74.38	TRUE	1.77E-22
CD4 on HLA DR+ CD4+ T cell	Wald ratio	1	1.63	1.32E-02	1.50E-04	2.46E-01	0.66	50.04	TRUE	4.35E-08
Effector memory CD8+ T cell %T cell	Inverse variance weighted	2	1.25	2.65E-02	1.56E-04	2.75E-01	0.33	51.15	TRUE	1.71E-17

R2.exposure and R2.outcome represent the phenotype variance which can be explained by the corresponding instrumental SNPs.

Among them, no exposure has instrumental SNPs of more than 2. Then the Wald ratio or IVW methods were used to conduct the MR analysis, and no proxy SNP was used in these exposures. Two instrumental SNPs (rs2571390, rs2523887) for exposure “CD8 on terminally differentiated CD8^+^ T cell” were not located on any known genes. Three SNPs corresponding to HVEM expression levels on T subsets were located on the HVEM encoding gene, TNFRSF14 perse (rs1886730, rs2227313), and a non-coding RNA gene LOC100996583 (rs2182176). One SNP was related to the exposure “CD4 regulatory T cell %T cell” and was located on splicing factor 45 encoding gene RBM17 (rs1571025). Notably, HVEM is a novel immune checkpoint that has never been linked with MG before. The SNPs found on the TNFRSF14 *per se* indicate a more direct link between the HVEM on T cells and MG.

### Secondary analysis: Validations in FIN and UK biobanks

**Figure 3:**
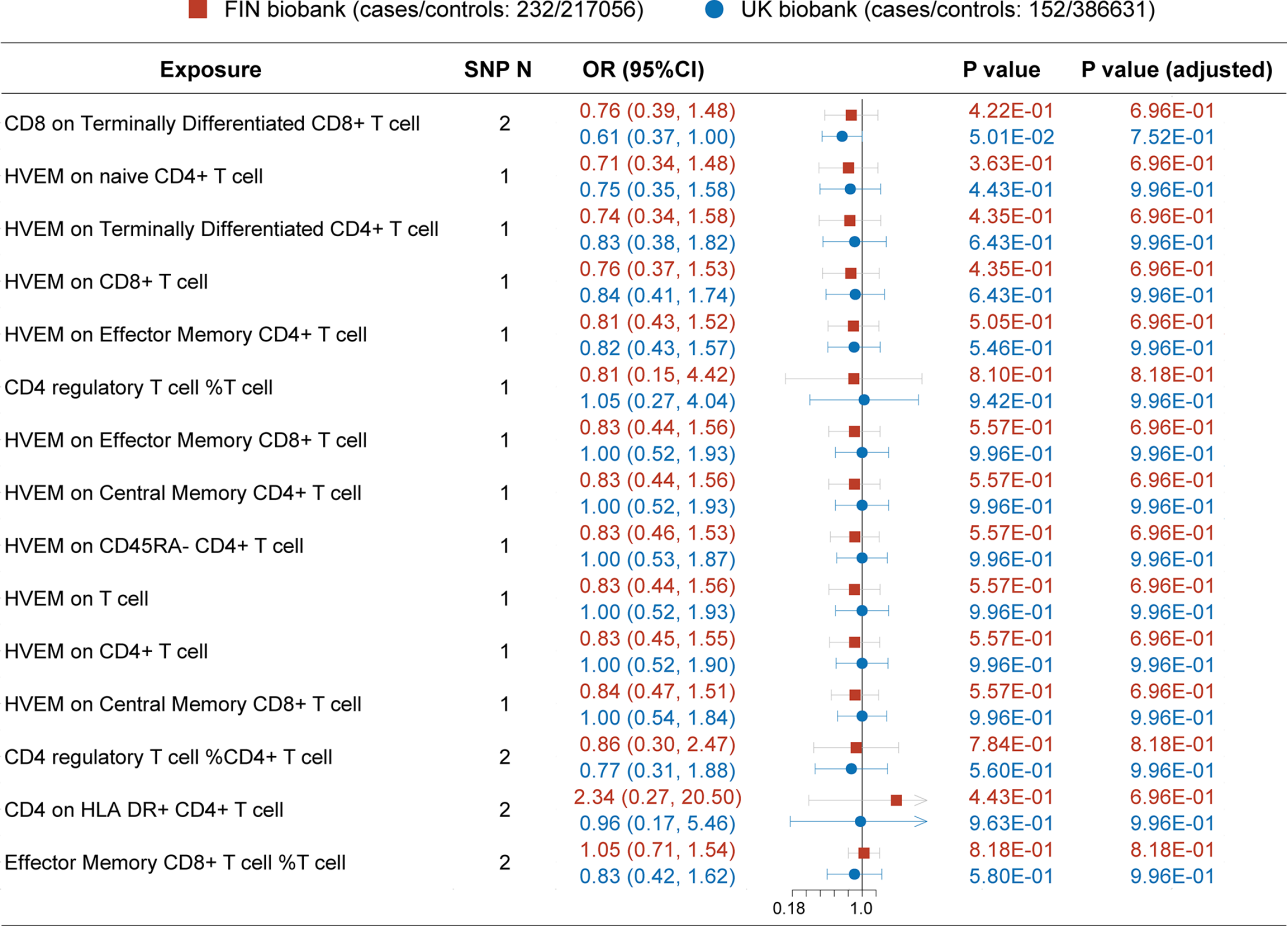
MR result in secondary analysis (FIN and UK Biobanks). Before P value adjustment, only the first ranked exposure “CD8 on terminally differentiated CD8^+^ T cell” barely showed significance in the UK biobank dataset. However, after FDR adjustment, no exposures reach significance, but the tendencies of which are basically in accordance with the primary analysis (as protective factors). This can be explained by the low powers in all analysis due to paucity in patients.

Since MG is a rare autoimmune disease with a low prevalence (around 12 per 100,000 population) (24), and another GWAS dataset with a large sample size of patients was not available, hence we conducted this replication in publicly available FIN and UK biobanks (**Figure 3**). Before P-value adjustment, the exposure “CD8 on Terminally Differentiated CD8^+^ T cell” in the UK biobank barely reached significance in MR analysis (OR [95% CI] = 0.61 [0.37, 1.00], P =5.01e-02), while after FDR adjustment, all results in both datasets showed a similar protective tendency with the primary analysis but did not reach significance. In the FIN biobank, the CD4^+^ regulatory T cell% T cell OR [95% CI] is 0.81 [0.15, 4.42], and HVEM on overall T cells is 0.83 [0.44, 1.56]. In the UK biobank, the CD4^+^ regulatory T cell% T cell OR [95% CI] is 1.05 [0.27, 4.04], and HVEM on overall T cells is 1.00 [0.52, 1.93]. This may be due to the much lower power in the FIN (median 0.09, IQR 0.04) and UK biobanks (median 0.03, IQR 0.04), as shown in **Tables 3**, **4**. Still, the Steiger directionality test showed that all results were consistent with the same exposure to outcome direction.

**Table 3:** Detailed MR result in the secondary analysis (FIN biobank).

Exposure	Method	SNP N	OR	r2.exposure	r2.outcome	P value (adjusted)	Power	F statistic	Correct causal direction	Steiger pval
CD8 on terminally differentiated CD8+ T cell	Inverse variance weighted	2	0.76	4.21E-02	7.75E-05	6.96E-01	0.13	82.46	TRUE	4.58031E-25
HVEM on naive CD4+ T cell	Wald ratio	1	0.71	3.30E-02	2.28E-05	6.96E-01	0.15	128.21	TRUE	5.39004E-10
HVEM on terminally differentiated CD4+ T cell	Wald ratio	1	0.74	2.81E-02	1.67E-05	6.96E-01	0.12	108.40	TRUE	1.07591E-08
HVEM on CD8+ T cell	Wald ratio	1	0.76	3.41E-02	1.67E-05	6.96E-01	0.12	132.69	TRUE	2.30064E-10
HVEM on effector memory CD4+ T cell	Wald ratio	1	0.81	4.15E-02	1.23E-05	6.96E-01	0.10	162.47	TRUE	1.89862E-12
CD4 regulatory T cell %T cell	Wald ratio	1	0.81	1.00E-02	1.59E-06	8.18E-01	0.05	37.96	TRUE	2.82692E-08
HVEM on effector memory CD8+ T cell	Wald ratio	1	0.83	4.03E-02	9.47E-06	6.96E-01	0.08	157.55	TRUE	3.68938E-12
HVEM on central memory CD4+ T cell	Wald ratio	1	0.83	4.25E-02	9.47E-06	6.96E-01	0.09	166.83	TRUE	8.65081E-13
HVEM on CD45RA- CD4+ T cell	Wald ratio	1	0.83	4.58E-02	9.47E-06	6.96E-01	0.09	180.31	TRUE	1.0565E-13
HVEM on T cell	Wald ratio	1	0.83	4.06E-02	9.47E-06	6.96E-01	0.08	158.78	TRUE	3.0409E-12
HVEM on CD4+ T cell	Wald ratio	1	0.83	4.44E-02	9.47E-06	6.96E-01	0.09	174.38	TRUE	2.66275E-13
HVEM on central memory CD8+ T cell	Wald ratio	1	0.84	4.93E-02	9.47E-06	6.96E-01	0.09	194.87	TRUE	1.09886E-14
CD4 regulatory T cell %CD4+ T cell	Inverse variance weighted	2	0.86	2.35E-02	2.17E-06	8.18E-01	0.05	45.13	TRUE	1.04689E-17
CD4 on HLA DR+ CD4+ T cell	Inverse variance weighted	2	2.34	2.47E-02	2.76E-04	6.96E-01	0.53	47.52	TRUE	5.01601E-14
Effector memory CD8+ T cell %T cell	Inverse variance weighted	2	1.05	2.65E-02	7.35E-06	8.18E-01	0.03	51.15	TRUE	1.54993E-19

**Table 4:** Detailed MR result in the secondary analysis (UK biobank).

Exposure	Method	SNP N	OR	r2.exposure	r2.outcome	P value (adjusted)	Power	F statistic	Correct causal direction	Steiger pval
CD8 on terminally differentiated CD8+ T cell	Inverse variance weighted	2	0.61	4.21E-02	1.09E-05	7.52E-01	0.24	82.46	TRUE	3.72264E-28
HVEM on naive CD4+ T cell	Wald ratio	1	0.75	3.30E-02	1.52E-06	9.96E-01	0.10	128.21	TRUE	1.30105E-10
HVEM on terminally differentiated CD4+ T cell	Wald ratio	1	0.83	2.81E-02	5.54E-07	9.96E-01	0.06	108.40	TRUE	3.14977E-09
HVEM on CD8+ T cell	Wald ratio	1	0.84	3.41E-02	5.54E-07	9.96E-01	0.06	132.69	TRUE	5.56983E-11
HVEM on effector memory CD4+ T cell	Wald ratio	1	0.82	4.15E-02	9.42E-07	9.96E-01	0.07	162.47	TRUE	4.52695E-13
CD4 regulatory T cell %T cell	Wald ratio	1	1.05	1.00E-02	1.36E-08	9.96E-01	0.03	37.96	TRUE	4.94225E-09
HVEM on effector memory CD8+ T cell	Wald ratio	1	1.00	4.03E-02	5.05E-11	9.96E-01	0.03	157.55	TRUE	7.87879E-13
HVEM on central memory CD4+ T cell	Wald ratio	1	1.00	4.25E-02	5.05E-11	9.96E-01	0.03	166.83	TRUE	1.72983E-13
HVEM on CD45RA- CD4+ T cell	Wald ratio	1	1.00	4.58E-02	5.05E-11	9.96E-01	0.03	180.31	TRUE	1.92226E-14
HVEM on T cell	Wald ratio	1	1.00	4.06E-02	5.05E-11	9.96E-01	0.03	158.78	TRUE	6.43707E-13
HVEM on CD4+ T cell	Wald ratio	1	1.00	4.44E-02	5.05E-11	9.96E-01	0.03	174.38	TRUE	5.04944E-14
HVEM on central memory CD8+ T cell	Wald ratio	1	1.00	4.93E-02	5.05E-11	9.96E-01	0.03	194.87	TRUE	1.80808E-15
CD4 regulatory T cell %CD4+ T cell	Inverse variance weighted	2	0.77	2.35E-02	2.81E-06	9.96E-01	0.07	45.13	TRUE	4.98314E-19
CD4 on HLA DR+ CD4+ T cell	Inverse variance weighted	2	0.96	2.47E-02	1.11E-05	9.96E-01	0.03	47.52	TRUE	1.3121E-17
Effector memory CD8+ T cell %T cell	Inverse variance weighted	2	0.83	2.65E-02	9.55E-06	9.96E-01	0.06	51.15	TRUE	5.80203E-21

## Discussion

This is the first MR study exploring the causal effects of risk factors on MG to the best of our knowledge. MR uses genetic variants as instrumental variables, fixed at conception, to conduct causal inferences about the impact of modifiable risk factors, which can overcome some types of confounding (25). This study was reported in accordance with the Strengthening the Reporting of Observational Studies in Epidemiology Using Mendelian Randomization (STROBE-MR) Statement (26). Our primary analysis extensively evaluated the causality between T-cell traits and MG, and three protective factors were identified in our study.

The first trait is the higher CD8 expression on terminally differentiated CD8^+^ T cells, the most mature CD8^+^ T cells residing in the periphery. Previous studies found that CD8 expression levels were lower in CD8^+^ T cells of chronic graft-versus-host disease and terminally differentiated effector memory T-cell (TEMRA) autoimmune lymphoproliferative syndrome (27, 28). CD8 is a coreceptor for the antigen-presenting process when activating T cells, and its downregulation on tissue-resident T cells has been postulated as a natural desensitization mechanism for prolonged antigen activation (29), which is common in the context of MG *per se* and its comorbidity with other autoimmune diseases (30). Higher CD8 expression levels on terminally differentiated CD8+ T cells represent an inert activated status. These inert CD8+ T cells are less likely to be activated by MG-related autoantigens, hence a less likely inclination to develop MG.

The second protective trait is a higher proportion of CD4^+^ Tregs, which is in accordance with previous studies. Previous GWASs on MG have identified the correlations between variants in genes (e.g., CTLA4 and PTPN22) with MG risk, which directly modulates the proportion or function of CD4^+^ Tregs (9, 11). Biological evidence from experimental autoimmune MG (EAMG) models has explained the potential mechanisms in which CD4^+^ Tregs suppressed the abnormal proliferation of T effector cells in response to MG-related antigens (31, 32). Our MR analysis validated the causality between CD4^+^ Tregs and MG, which supported the hypothesis that individuals with more CD4^+^ Tregs would be less likely to develop MG.

Interestingly, the third protective trait is the higher HVEM expression on various T-cell subtypes. HVEM, which belongs to the tumor necrosis factor receptor (TNFR) superfamily, has been recognized as a novel immune checkpoint in recent years (33). HVEM is expressed primarily on immune cells and functions as a ligand to activate the B- and T-lymphocyte attenuator (BTLA) on other immune cells (34). Two categories of BTLA are CTLA-4/CD28/CD80/CD86 (function at the early phase of T-cell activation) and PD-1/PD-L1/PD-L2 (control the effector phase of the immune response in peripheral tissues) (35). The former (CTLA-4) expression has been found lower in MG patients, and the latter (PD-1) has been linked with immune checkpoint inhibitor-related myasthenia gravis (36, 37). As an immune inhibiting ligand, higher HVEM expression on T cells may be a protective factor for MG. The other function of HVEM is that it mediates the entry of herpes simplex virus type 1 (HSV-1) and HSV-2 into cells, which do not include other subtypes such as Epstein–Barr (EB) virus and varicella zoster virus (VZV) (38). We think that this might explain why fewer HSV-infected MG patient cases were reported than those EB and VZV cases in clinical settings (39, 40). However, studies with larger sample size and stringent design are needed to validate this in future.

Noted that MG is a rare neuromuscular disease; the sample size derived from now available GWAS datasets is still not satisfactory for data-driven analysis. However, we attempted to replicate the findings in another two independent biobanks. In the replication process, only similar protective tendencies, albeit not significant, were found in these exposures, which is restrained by the small power due to paucity in patients. Given that our results can explain the potential biological mechanism underlying T cells in MG genesis, this MR analysis basically satisfied the required assumptions in MR studies (relevance, independence, and exclusion restriction) (25).

There are several limitations in this study: 1) The primary results were derived from AChR+ MG patients, and the secondary results derived from MG with unknown subtypes. Hence, caution is needed to interpret the results. 2) There is insufficient validation in large exposure and outcome datasets. 3) The participants of the FIN and UK biobanks were enrolled by self-reported results, which may introduce biases in the results. 4) Horizontal pleiotropy was found in selected SNPs with other autoimmune diseases, which may interfere with MG pathogenesis by other immunological pathways, not only through T cells. 5) The ancestry of GWAS data used in this study is mainly of European origin, and further GWASs from other races are needed to validate the results.

## Conclusions

In conclusion, we found three T-cell-related traits as potential protective factors for the risk of MG in the primary analysis: 1) CD8 on terminally differentiated CD8^+^ T cells, 2) CD4^+^ regulatory T cell% T cells, and 3) HVEM on overall T cells. In the future, these factors may serve as biomarkers for forecasting MG development and provide new insights into the underlying mechanism.

## Data availability statement

The original contributions presented in the study are included in the article/**Supplementary Material**. Further inquiries can be directed to the corresponding authors.

## Ethics statement

The studies involving human participants were reviewed and approved by the ethical committees of the original GWAS studies analyzed in this study. All original studies have obtained ethical approval and informed consent from the participants. Written informed consent to participate in this study was provided by the participants’ legal guardian/next of kin.

## Author contributions

SL and CZ conceived the presented idea. HZ, KJ, and XH performed the computations and manuscript writing. MS, XZ, and ZZ were involved in interpretation of data. All authors contributed to the article and approved the submitted version.

## Funding

This work has been supported by grants from China’s National Natural Science Foundation (Nos. 81870988, 82071410, and 82001335) and the Shanghai Municipal Science and Technology Major Project (No. 2018SHZDZX01), and ZJLab.

## Acknowledgments

The authors thank all investigators of the three GWAS summary datasets used in this study, for sharing them publicly for research. We also want to thank Dr. Shuyi Huang (Huashan Hospital) for providing insightful suggestions on the MR methodology of this study.

## Conflict of interest

The authors declare that the research was conducted in the absence of any commercial or financial relationships that could be construed as a potential conflict of interest.

## Publisher’s note

All claims expressed in this article are solely those of the authors and do not necessarily represent those of their affiliated organizations, or those of the publisher, the editors and the reviewers. Any product that may be evaluated in this article, or claim that may be made by its manufacturer, is not guaranteed or endorsed by the publisher.

## Supplementary material

Supplementary File 1 The detailed instrumental SNPs information of primary and secondary analysis.

Supplementary File 2 The detailed MR results of all exposures in primary and secondary analysis.

Supplementary File 3 Pleiotropy analysis results for those significant SNPs displayed in primary and secondary analysis.
